# Vitamin D Supplementation Enhances C18(dihydro)ceramide Levels in Type 2 Diabetes Patients

**DOI:** 10.3390/ijms18071532

**Published:** 2017-07-15

**Authors:** Alexander Koch, Georgios Grammatikos, Sandra Trautmann, Yannick Schreiber, Dominique Thomas, Franziska Bruns, Josef Pfeilschifter, Klaus Badenhoop, Marissa Penna-Martinez

**Affiliations:** 1 Department of General Pharmacology and Toxicology, Goethe University Hospital, 60590 Frankfurt am Main, Germany; georgios.grammatikos@kgu.de (G.G.); pfeilschifter@em.uni-frankfurt.de (J.P.); 2 Department of Medicine I, Goethe University Hospital, 60590 Frankfurt am Main, Germany; 3 Department of Clinical Pharmacology, Goethe University Hospital, 60590 Frankfurt am Main, Germany; labocha@med.uni-frankfurt.de (S.T.); thomas@med.uni-frankfurt.de (D.T.); 4 Fraunhofer Institute of Molecular Biology and Applied Ecology—Project Group Translational Medicine and Pharmacology (IME-TMP), 60590 Frankfurt am Main, Germany; schreiber@med.uni-frankfurt.de; 5 Department of Internal Medicine I, Division of Endocrinology, Diabetes and Metabolism, Goethe University Hospital, 60590 Frankfurt am Main, Germany; franziskabruns85@gmail.com (F.B.); badenhoop@em.uni-frankfurt.de (K.B.); Marissa.Penna-Martinez@kgu.de (M.P.-M.)

**Keywords:** vitamin D, Type 2 Diabetes mellitus, sphingolipid metabolism, ceramide, dihydroceramide, sphingosine 1-phosphate

## Abstract

Sphingolipids are characterized by a broad range of bioactive properties. Particularly, the development of insulin resistance, a major pathophysiological hallmark of Type 2 Diabetes mellitus (T2D), has been linked to ceramide signaling. Since vitamin D supplementation may slow down T2D progression by improving glucose concentrations and insulin sensitivity, we investigated whether vitamin D supplementation impacts on plasma sphingolipid levels in T2D patients. Thus, plasma samples of 59 patients with non-insulin-requiring T2D from a placebo-controlled, randomized, and double-blind study were retrospectively analyzed. Once per week, patients received either 20 drops of Vigantol oil, corresponding to a daily dose of 1904 IU/d vitamin D (verum: *n* = 31), or a placebo oil consisting of medium chain triglycerides (placebo: *n* = 28). Blood samples were taken from all of the participants at three different time points: 1) at the beginning of the study (baseline), 2) after 6 months supplementation, and 3) after an additional 6 months of follow-up. Plasma sphingolipids were measured by high-performance liquid chromatography tandem mass spectrometry. At baseline and 6 months follow-up, no significant differences in plasma sphingolipid species were detected between the placebo and verum groups. After 6 months, vitamin D supplementation significantly enhanced plasma C18dihydroceramide (dhCer; *N*-stearoyl-sphinganine (d18:0/18:0)) and C18ceramide (Cer; *N*-stearoyl-sphingosine (d18:1/18:0)) levels were observed in the verum group compared to the placebo group. This was accompanied by significantly higher 25-hydroxyvitamin D_3_ (25(OH)D_3_) blood levels in patients receiving vitamin D compared to the placebo group. Taken together, vitamin D supplementation induced changes of the C18 chain-length-specific dhCer and Cer plasma levels in patients with T2D. The regulation of sphingolipid signaling by vitamin D may thus unravel a novel mechanism by which vitamin D can influence glucose utilization and insulin action. Whether this acts favorably or unfavorably for the progression of T2D needs to be clarified.

## 1. Introduction

Today, globally, more than 415 million patients suffer from diabetes mellitus, one of the major health problems worldwide with increasing prevalence. By 2040, one in ten adults will have diabetes. Almost 90% of all patients will have Type 2 Diabetes mellitus (T2D), the most prevalent form in industrial countries (International Diabetes Federation, “Diabetes Atlas—the 7th edition”). The development of T2D is believed to be influenced by a combination of genetic background and multiple environmental factors, among which vitamin D status has been identified [1]. The active form of vitamin D, 1α,25-dihydroxyvitamin D_3_ (1,25(OH)_2_D_3_ vitamin D, calcitriol), is synthesized from cholesterol through a photochemical reaction to sunlight in the skin, followed by enzymatic conversions in the liver and kidney. Although it can be taken up partially by food, synthesis due to natural sunlight exposure in the skin remains the major source of vitamin D, which explains why vitamin D deficiency is a common problem in healthy humans [2]. Since it is more stable and highly abundant in blood, the first metabolite 25-hydroxyvitamin D_3_ (25(OH)D_3_) is normally used to determine the vitamin’s status in human blood. Vitamin D deficiency is defined by 25(OH)D_3_ blood levels less than 20 ng/mL, and was found to be associated with an increasing risk for numerous diseases including hypertension, obesity, and diabetes [2,3]. In this context, several studies revealed that patients with T2D have lower levels of 25(OH)D_3_, which correlates with a higher risk for the development of T2D [4,5,6]. On the other hand, vitamin D supplementation may slow down T2D progression by improving glucose concentrations and insulin sensitivity [7,8,9]. Mechanistically, vitamin D can influence the insulin sensitivity directly by (1) the stimulation of the expression of insulin receptor and (2) the activation of peroxisome proliferator-activated receptor delta or (3) indirectly via the regulation of calcium homeostasis [10].

Interestingly, the development of insulin resistance, a major pathophysiological hallmark of T2D, has been linked to sphingolipid signaling [11,12]. Sphingolipids consist of more than 300 structural diverse molecules with a broad range of bioactive properties. Various members of this special class of lipids were identified as important mediators in the pathogenesis of several diseases [13,14]. Particularly, the role of ceramide for the progression of diabetes mellitus was extensively investigated in the last years. In general, ceramide inhibits signaling pathways downstream of the insulin receptor, e.g., insulin receptor substrate 1 phosphorylation, activation of phosphatidylinositol 3-kinase, and Akt/PKB, and thereby blocks the translocation of glucose transporter GLUT4 and induces pancreatic β-cell apoptosis [11,15]. In line with these findings, different studies revealed that the accumulation of ceramide occurs in the skeletal muscle of insulin-resistant humans [16], which negatively correlates with insulin sensitivity [17]. However, another study failed to demonstrate enhanced muscle ceramide levels in insulin resistant and T2D patients, fostering ongoing discussions on its contribution to insulin resistance [18,19].

In the current study, we investigated whether vitamin D supplementation influences the plasma content of long chain and very long chain ceramides and their precursor’s dihydroceramides, as well as the degradation products sphinganine, sphingosine, and their 1-phosphate derivatives. To our knowledge, this is the first report investigating a possible link between vitamin D uptake and changes in plasma sphingolipid levels in humans.

## 2. Results

### 2.1. Clinical and Biochemical Parameters

The characteristics of all patients (placebo group: 28 (13 male/15 female), verum group: 31 (16 male/15 female)) included in this retrospective analysis are shown in Table 1. The median age of the patients in the placebo group was 60 years and in the verum group 62 years with a median diabetes duration of 7 and 5 years, respectively. The median body mass index (BMI) was 31 kg/m^2^ in both groups. As shown in Table 2, the blood cholesterol, high density lipoprotein (HDL)-cholesterol, low density lipoprotein (LDL)-cholesterol, and triglyceride levels were not significantly different between the placebo and verum groups at baseline. Further, treatment with 1904 IU/d vitamin D for 6 months did not influence the amounts of total cholesterol, HDL-cholesterol, LDL-cholesterol, and triglycerides in blood compared to the placebo-treated group (Table 2).

According to the previous publication [20], all patients included in the current analysis showed at baseline a vitamin D deficiency with 25(OH)D_3_ concentrations of 12 (8.6–15) ng/mL in the placebo group and 13 (8.7–18) ng/mL in the verum group. After 6 months of vitamin D supplementation, the patients in the verum group had significantly higher 25(OH)D_3_ levels compared to the placebo group (placebo group: 11 (7.2–16) ng/mL, verum group: 29 (21–32) ng/mL, *p* < 0.0001). After an additional 6 months of follow-up, the 25(OH)D_3_ levels were still significantly elevated in the verum group compared to the placebo group (placebo group: 12 (9.9–16) ng/mL, verum group: 19 (15–22) ng/mL, *p* = 0.0007).

### 2.2. Plasma Sphingolipid Levels

**Figure 1:**
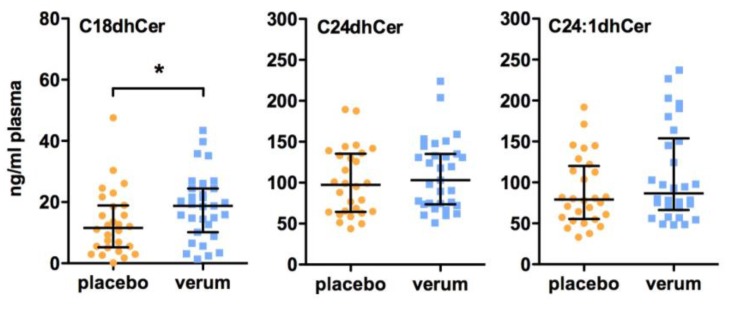
Effect of vitamin D supplementation on plasma dihydroceramide (dhCer) levels. All patients were treated for 6 months with either a placebo or 1904 IU/d vitamin D. Plasma sphingolipid concentrations were measured by LC-MS/MS. Dates are shown as median ± IQR (Mann Whitney U test, * *p* < 0.05). Abbreviations: dhCer, dihydroceramide.

**Figure 2:**
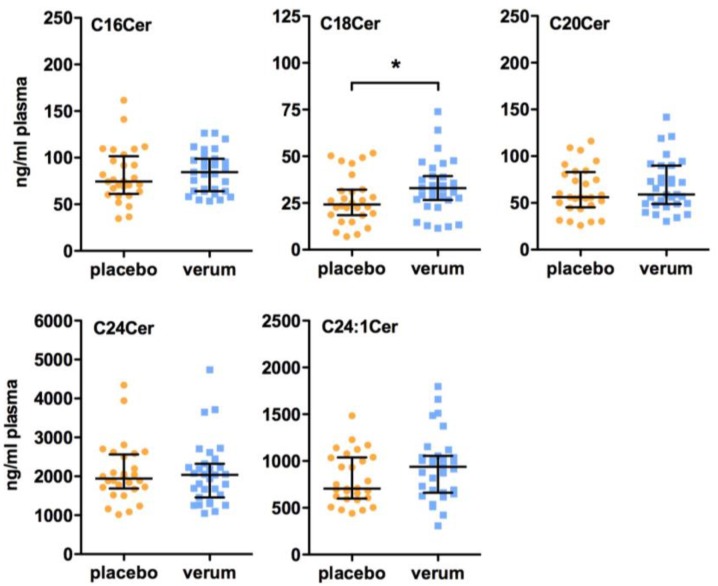
Effect of vitamin D supplementation on plasma ceramide (Cer) levels. All patients were treated for 6 months with either a placebo or 1904 IU/d vitamin D. Plasma sphingolipid concentrations were measured by LC-MS/MS. Dates are shown as median ± IQR (Mann Whitney U test, * *p* < 0.05). Abbreviations: Cer, ceramide.

Here, we could show by LC-MS/MS analysis that the plasma concentrations of chain-length specific ceramides and dihydroceramides, bioactive synthetic precursors of ceramides, in patients suffering from T2D were influenced by vitamin D supplementation. After 6 months of vitamin D treatment, plasma levels of C18dhCer were significantly elevated in the verum group compared to the placebo group (placebo group: 11.6 (5.21–18.9) ng/mL, verum group: 18.8 (10.2–24.5) ng/mL, *p* = 0.036; Figure 1). In line with this, the subsequent C18Cer levels were also significantly higher in the vitamin D-treated group compared to the placebo group (placebo group: 24.3 (18.5–32.1) ng/mL, verum group: 33.0 (26.7–39.4) ng/mL, *p* = 0.040; Figure 2). At baseline, C18dhCer and C18Cer were similar in the placebo and verum groups (C18dhCer: placebo group: 14.5 (8.86–33.3) ng/mL, verum group: 17.3 (13.6–25.2) ng/mL, *p* = 0.250 (missing data: *n* = 27 (placebo group), *n* = 30 (verum group); C18Cer: placebo group: 30.3 (24.0–46.1) ng/mL, verum group: 36.0 (24.7–39.4) ng/mL, *p* = 0.856). Interestingly, after 6 months follow-up, no significant differences in C18dhCer and C18Cer levels were detected between the placebo group and the verum group (C18dhCer: placebo group: 9.16 (3.82–23.5) ng/mL, verum group: 20.1 (5.60–26.3) ng/mL, *p* = 0.170; C18Cer: placebo group: 21.4 (10.6–32.0) ng/mL, verum group: 23.1 (18.4–35.9) ng/mL, *p* = 0.211).

**Figure 3:**
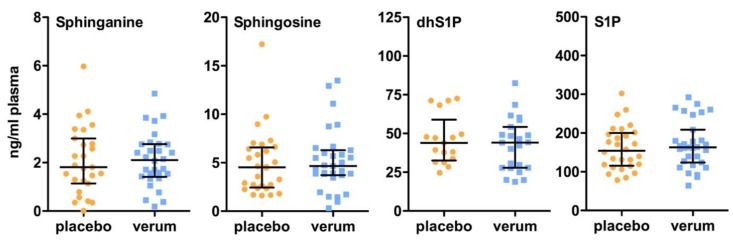
Effect of vitamin D supplementation on plasma sphinganine, sphingosine, and 1-phosphate derivate levels. All patients were treated for 6 months with either a placebo or 1904 IU/d vitamin D. Plasma sphingolipid concentrations were measured by LC-MS/MS. Dates are shown as median ± IQR (Mann Whitney U test). Abbreviations: dhS1P, sphinganin 1-phosphate; S1P, sphingosine 1-phosphate.

As illustrated in Figure 1 and Figure 2, all other dihydroceramides and ceramides remain unchanged after 6 months of vitamin D supplementation. Moreover, no differences between the placebo group and the verum group were observed in the plasma levels of sphingosine, sphinganine, and their 1-phosphate derivatives (Figure 3). In line with these findings, no significant differences in the plasma levels of all of the other dihydroceramides and ceramides, sphingosine, sphinganine, dhS1P, and S1P were present between the placebo and verum groups at baseline (Table S1) and 6 months follow-up after vitamin D supplementation (Table S2).

## 3. Discussion

Beside the well-known effects on bone formation and mineralization [21], there is growing evidence for pleiotropic actions of vitamin D via the vitamin D receptor (VDR) that is expressed abundantly [22]. With regards to T2D, these extraskeletal effects are particularly evident for the immune system and endothelial function, with controversial discussions about the clinical studies [23]. Nevertheless, several studies showed beneficial effects of vitamin D on glucose status, as well as on insulin sensitivity and resistance [7,8,9]. In line with these findings, we could previously show that vitamin D supplementation enhanced 25OHD in plasma levels, which was associated, e.g., with significantly lower HbA1c levels [20]. Vitamin D supplementation may therefore stabilize glucose homeostasis and help to slow the progression of T2D [20]. Here, we used a total number of 59 plasma samples from our previous study to investigate retrospectively whether the uptake of 1904 IU/d vitamin D for 6 months had an impact on plasma sphingolipid levels in patients suffering from T2D. Several studies reveal that sphingolipids such as ceramide influence metabolic pathways and the cells involved in the development of diabetes [11]. However, to our knowledge, there has not been any study yet about vitamin D’s effects on sphingolipid metabolism in diabetes patients. By using LC-MS/MS analyses, we observed that the levels of both C18Cer and its precursor C18dhCer were significantly enhanced by vitamin D supplementation compared to the placebo group. All of the other dihydroceramides and ceramides as well as their degradation products remain unchanged by vitamin D supplementation. In general, C18dhCer and C18Cer concentrations are known to be in a lower micromolar range in plasma and serum compared to very long-chain ceramides [24], which is contrary in tissues such as skleletal muscle [25]. However, the potential of single ceramides to influence the progression of T2D seems not to be concentration but chain-length dependent. Overall, there is strong evidence that ceramide species exert different effects depending on the chain-lengths of the fatty acid bound to the sphingosine backbone. For example, C16Cer and C18Cer induce cell death, whereas very long chain C24Cer and C24:1Cer mediate anti-apoptotic effects [26,27]. In the context of T2D development, Sugimoto et al. [28] suggested that enhanced plasma levels of very long chain ceramides contribute to the elevated glucose clearance observed in sphingomyelin synthase 2 knockout mice. The overexpression of CerS2, necessary for the formation of very long chain ceramides (C20Cer/C24Cer/C24:1Cer) [27], led to an improvement in insulin signal transduction, as well as decreased endoplasmic reticulum stress and markers for gluconeogenesis in mouse hepatocytes [29]. On the other hand, the lack of CerS2, and the subsequent elimination of these ceramide species causes hepatic insulin resistance in mice [30]. Overall, these data reveal a protective role of long chain ceramides against the development of glucose intolerance and hepatic insulin resistance. In many studies, elevated short chain ceramide levels have been linked to insulin resistence and the development of T2D. For example, Haus et al. [31] showed that enhanced plasma ceramide levels in patients with T2D correlate with the severity of insulin resistance. The authors suggested that the activation of tumor necrosis factor-alpha by short chain C18Cer and C18:1Cer may contribute to insulin resistance in patients [31]. In addition, it was shown that glucolipotoxicity induces β-cell apoptosis through the formation of ceramides with specific N-acyl chain lengths, such as C18Cer and its corresponding dihydroceramides [32]. Most interestingly, in a recent publication Wigger et al. [33] demonstrated elevated dihydroceramide levels in individuals who will progress to diabetes up to 9 years before the disease’s onset. Therefore, the authors suggested that these lipids may serve as early biomarkers for T2D development. The authors also showed that ceramides/dihydrceramides positively correlate with glucose intolerance and negatively correlate to insulin sensitivity [33]. From our data, we can neither confirm nor exclude that the observed changes in C18dhCer and C18Cer upon vitamin D supplementation can influence glucose uptake, insulin resistance, and thereby the course of T2D favorably or unfavorably. Thus, further studies need to answer by which mechanism vitamin D supplementation influences T2D progression. One of the mechanisms may include elevated C18dhCer and C18Cer levels. In order to elucidate the mechanisms, we would dissect whether vitamin D can activate specific CerS. CerS2 and 4 are the most prominent isoforms in blood, which catalyze the formation of either C20-26Cer (CerS2) or C18/20Cer (CerS4), as well as their dihydroceramide analogs [27]. To date, there is no evidence from the literature that vitamin D can influence specific CerS isoforms in order to generate chain length-specific ceramides and dihydroceramides. However, since we found out that vitamin D selectively enhanced C18(dh)Cer levels in T2D patients’ plasma, CerS4 seems to be an attractive target for future in vitro and in vivo studies. Interestingly, recent publications shed new light on the shingolipids´ potential in T2D prediction. Wigger et al. [33] showed the effect of vitamin D uptake on C18dhCer and C18Cer levels at the onset of T2D in humans. A large population-based study of individuals, some of whom later developed T2D [34], shows a strong predictive power for 1-deoxySL levels, indicative of T2D development in the next 5 years, particularly in non-obese individuals. Together with our observations, this illustrates the so far neglected potential of sphingolipid markers in T2D.

## 4. Materials and Methods

### 4.1. Patients and Sampling

In the current study, we evaluated retrospectively the effect of vitamin D supplementation on plasma sphingolipid levels of patients with noninsulin-requiring T2D from the city of Frankfurt am Main (Germany). Overall, plasma samples of 59 patients from a placebo-controlled, randomized, and double-blind study were analyzed [20]. The study was performed in accord with the Declaration of Helsinki and was approved by the Ethics Committee of Frankfurt University Hospital (21 January 2008, Reference number: 9/07, Protocoll number: EMD 28162-600, EudraCT: 2006-006180-23). All patients had provided written informed consent before their inclusion in the study. All participants were not treated with bisphosphonates, calcimimetics, glucocorticoids, phenytoin, glycosides, or benzodiazepine, before or during the study period. Further, all patients did not receive vitamin D supplementation for at least 3 months before the beginning of the study (baseline). At baseline, patients’ characteristics (age, gender, duration of T2D, body mass index (BMI)) were determnined and the participants were randomized into two parallel groups. During 6 months, patients received once weekly either 20 drops of Vigantol oil, corresponding to a daily dose of 1904 IU/d vitamin D (verum group: *n* = 31), or a placebo oil consisting of medium chain triglycerides (placebo group: *n* = 28) at baseline. Blood laboratory parameters (triglycerides, cholesterol, low density lipoproteins (LDL), high density lipoproteins (HDL)) were determined at baseline and after 6 months of supplementation. For the sphingolipid and 25(OH)D_3_ analysis at all timepoints, blood samples (1.6 mg/mL ethylenediamine tetraacetic acid (EDTA) as anticoagulant) were centrifuged at room temperature for 10 min at 400× *g* and plasma aliquots from each subject were immediately frozen at −80 °C until assayed. The concentrations of 25(OH)D_3_ were measured by ^125^I-radioimmunoassay. The plasma sphingolipid levels were measured by high-performance liquid chromatography/tandem mass spectrometry (LC-MS/MS) as described below.

### 4.2. LC-MS/MS Analysis

For lipid extraction, 10 µL of plasma was mixed with 150 µL of water, 150 µL of extraction buffer (citric acid 30 mM, disodium hydrogen phosphate 40 mM), and 20 µL of the internal standard solution containing sphingosine-d7, sphinganine-d7 (200 ng/mL each), sphingosine-1-phosphate-d7, C17:0 Cer, C16:0 Cer-d31, C18:0 Cer-d3, C17:0 LacCer, C18:0 DHC-d3, C16:0 LacCer-d3, C18:0 GluCer-d5 (all Avanti Polar Lipids, Alabaster, AL, USA), and C24:0 Cer-d4 (Chiroblock GmbH, Bitterfeld-Wolfen, Germany; 400 ng/mL each). The mixture was extracted once with 1000 µL methanol/chloroform/hydrochloric acid (15:83:2, *v*/*v*/*v*). The lower organic phase was evaporated at 45 °C under a gentle stream of nitrogen and reconstituted in 200 µL of tetrahydrofuran/water (9:1, *v*/*v*) with 0.2% formic acid and 10 mM ammonium formate. Afterwards, the amounts of sphingolipids were analyzed by liquid chromatography coupled to tandem mass spectrometry (LC-MS/MS). An Agilent 1100 series binary pump (Agilent technologies, Waldbronn, Germany) equipped with a Luna C8 column (150 mm × 2 mm ID, 3 μm particle size, 100 Å pore size; Phenomenex, Aschaffenburg, Germany) was used for chromatographic separation. The column temperature was 35 °C. The HPLC mobile phases consisted of water with 0.2% formic acid and 2 mM ammonium formate (mobile phase A) and acetonitrile/isopropanol/acetone (50:30:20, *v*/*v*/*v*) with 0.2% formic acid (mobile phase B). For separation, a gradient program was used at a flow rate of 0.3 mL/min. The initial buffer composition 55% (A)/45% (B) was held for 0.7 min and then within 4 min linearly changed to 0% (A)/100% (B) and held for 13.3 min. Subsequently, the composition was linearly changed within 1 min to 75% (A)/25% (B) and then held for another 2 min. The total running time was 21 min, and the injection volume was 15 μL. To improve ionization, acetonitrile with 0.1% formic acid was infused post-column using an isocratic pump at a flow rate of 0.15 mL/min. After every sample, sample solvent was injected for washing the column with a 12 min run. The MS/MS analyses were performed using a triple quadrupole mass spectrometer API4000 (Sciex, Darmstadt, Germany) equipped with a Turbo V Ion Source operating in positive electrospray ionization mode. The MS parameters were set as follows: Ionspray voltage 5500 V, ion source temperature 500 °C, curtain gas 30 psi, collision gas 12 psi, nebulizer gas 40 psi, and heating gas 60 psi. The analysis was done in Multiple Reaction Monitoring (MRM) mode.

Data Acquisition was done using Analyst Software V 1.6, and quantification was performed with MultiQuant Software V 3.0 (both Sciex, Darmstadt, Germany), employing the internal standard method (isotope dilution mass spectrometry). Variations in accuracy of the calibration standards were less than 15% over the whole range of calibration, except for the lower limit of quantification, where a variation in accuracy of 20% was accepted.

### 4.3. Statistical Analysis

The statistical analyses were performed with GraphPad Prism (v5.01; GraphPad Software Inc., San Diego, CA, USA). Group comparisons were conducted by using the nonparametric Mann Whitney U test. Differences with *p* < 0.05 were considered to be significant. All data are expressed as median ± interquartile range (IQR).

## 5. Conclusions

Taken together, we demonstrate here for the first time that vitamin D supplementation enhances the plasma levels of C18dhCer and C18Cer in patients suffering from T2D. These findings offer promising new approaches to understand the role of vitamin D for the development of Diabetes mellitus by influencing sphingolipid metabolism.

## Figures and Tables

**Table 1:** Patients’ characteristics at baseline.

Patients’ Characteristics	Placebo	Verum	*p*-Value
Age (years)	60 (52–66)	62 (54–67)	0.395
BMI (kg/m^2^)	31 (27–35)	31 (27–33)	0.791
Duration of T2D (years)	7 (4–10)	5 (3–9)	0.273

Median (interquartile range, IQR); Mann Whitney U test. Abbreviations: BMI, body mass index; T2D, Type 2 Diabetes mellitus.

**Table 2:** Blood lipid levels at baseline and after 6 months of supplementation.

Lipid Profile	Baseline			After 6 Months Supplementation		
	Placebo	Verum	*p*-Value	Placebo	Verum	*p*-Value
Triglycerides	143 (104–222)	151 (98–185)	0.750	147 (111–198)	145 (100–224)	0.994
Cholesterol	194 (181–211)	198 (174–227)	0.738	200 (171–229)	205 (176–231)	0.733
LDL-cholesterol	109 (98–135)	123 (97–144)	0.275	118 (102–137)	133 (96–145)	0.524
HDL-cholesterol	46 (37–54)	47 (37–58)	0.837	48 (41–58)	50 (40–56)	0.727

Median (IQR); mg/dl; *n* = 28 (placebo group), *n* = 31 (verum group); Mann Whitney U test; Missing data: LDL: *n* = 27 (placebo group at baseline). Abbreviations: HDL, high density lipoproteins; LDL, low density lipoproteins.

## Supplementary Materials

Supplementary materials can be found at www.mdpi.com/1422-0067/18/7/1532/s1.

## Abbreviations

25(OH)D_3_	25-hydroxyvitamin D_3_
Cer	ceramide
CerS	ceramide synthase
dhCer	dihydroceramide
HDL	high densitiy lipoproteins
LDL	low densitiy lipoprotein
dhS1P	sphinganine 1-phosphate
S1P	sphingosine 1-phosphate
T2D	Type 2 Diabetes mellitus
