# Supplementary material for: Vitamin D Supplementation Enhances C18(dihydro)ceramide Levels in Type 2 Diabetes Patients
Source: Int J Mol Sci. 2017 Jul 15;18(7):1532. doi: 10.3390/ijms18071532 (PMC5536020; doi:10.3390/ijms18071532)
Supplement: Supplementary file 1 [file ijms-18-01532-s001.pdf]

Vitamin D Supplementation Enhances C18(dihydro)ceramide Levels in Type 2 Diabetes Patients

Table S1. Plasma sphingolipid concentrations at baseline.

	Placebo	Verum	p-value
C24dhCer	115 (82.8-164)	116 (85.1-144)	0.611
C24:1dhCer	91.4 (63.4-126)	103 (71.0-125)	0.462
C16Cer	86.1 (62.0-99.5)	80.3 (66.8-95.8)	0.958
C20Cer	65.5 (45.0-96.9)	62.8 (47.3-111)	0.633
C24Cer	2447 (1845-2929)	2173 (1691-2410)	0.091
C24:1Cer	793 (702-1265)	858 (728-1076)	0.982
Sphinganine	2.11 (1.07-3.21)	2.18 (1.44-2.85)	0.987
Sphingosine	5.47 (3.98-7.48)	5.24 (3.96-7.26)	0.826
dhS1P	34.0 (20.9-42.4)	34.1 (27.1-43.6)	0.643
S1P	236 (200-281)	211 (171-239)	0.070

Median (IQR); mg/ml; n = 28 (placebo group), n = 31 (verum group); Mann Whitney U test. Missing data: sphinganine: n = 27 (placebo group), n = 30 (verum group). Abbreviations: dhCer, dihydroceramide; Cer, ceramide; dhS1P, sphinganine 1-phosphate; S1P, sphingosine 1-phosphate.

Table S2. Plasma sphingolipid concentrations at follow-up.

	Placebo	Verum	p-value
C24dhCer	107 (74.1-154)	100 (77.3-157)	0.994
C24:1dhCer	78.1 (53.8-111)	80.9 (58.8-113)	0.519
C16Cer	65.5 (54.3-96.2)	77.4 (60.2-90.6)	0.351
C20Cer	56.1 (45.6-78.2)	69.0 (46.5-84.7)	0.200
C24Cer	2044 (1614-2412)	1838 (1531-2310)	0.539
C24:1Cer	632 (458-912)	691 (570-888)	0.452
Sphinganine	3.13 (2.42-4.36)	3.88 (2.77-4.79)	0.222
Sphingosine	7.74 (3.32-12.5)	9.22 (6.08-16.3)	0.375
dhS1P	42.7 (27.6-54.4)	36.0 (26.3-41.8)	0.174
S1P	196 (127-228)	172 (153-226)	0.814

Median (IQR); mg/ml; n = 28 (placebo group), n = 31 (verum group); Mann Whitney U test. Abbreviations: dhCer, dihydroceramide; Cer, ceramide; dhS1P, sphinganine 1-phosphate; S1P, sphingosine 1-phosphate.
